# The DID of CAPS-1 anchors plasma membrane to promote vesicle exocytosis

**DOI:** 10.1016/j.jbc.2025.110902

**Published:** 2025-11-04

**Authors:** Li Zhang, Yuwan Dai, Ting Sun, Wanhai Wang, Ziqing Wei

**Affiliations:** 1 Department of Clinical Laboratory, The First Affiliated Hospital of Zhengzhou University, Zhengzhou, Henan, China; 2 Department of Blood Transfusion, Henan Provincial People's Hospital, Zhengzhou, Henan, China; 3 Department of Neurology, The First Affiliated Hospital of Zhengzhou University, Zhengzhou, Henan, China

**Keywords:** Ca^2+^-dependent exocytosis, CAPS-1, DID, SNARE complex assembly, vesicle exocytosis

## Abstract

Ca^2+^-dependent activator proteins for secretion are multidomain proteins involved in Ca^2+^-regulated exocytosis of synaptic vesicles and dense core vesicles. Ca^2+^-dependent activator proteins for secretion contain a dynactin1-interacting domain (DID), previously implicated in vesicle sorting. Here, we reveal a novel role for the DID in direct membrane association. Using structural modeling, liposome binding, and fusion assays, we demonstrate that the DID binds to and clusters negatively charged phospholipids, such as phosphatidylserine and phosphatidylinositol 4,5-bisphosphate, and significantly enhances SNARE-mediated liposome fusion. Furthermore, deletion of the DID in PC12 cells impairs evoked vesicle release. Our findings establish the DID as a critical plasma membrane–anchoring module that promotes efficient vesicle exocytosis.

Intercellular communication depends on the release of neurotransmitters or neuropeptides by the dense core vesicles (DCVs) or synaptic vesicles (SVs) (1). This process involves several steps, including vesicle tethering to presynaptic active zones, docking to the phosphatidylinositol 4,5-bisphosphate (PI(4,5)P2)–enriched sites, priming for release, and eventual Ca^2+^-triggered membrane fusion (2, 3). Ca^2+^-dependent activator proteins for secretion (CAPS) play an essential role in all stages of vesicle release (4, 5, 6, 7). Deficiency of CAPS in *Caenorhabditis elegans*, *Drosophila*, or mammalian neurons results in impaired DCV exocytosis and reduced vesicle docking (8, 9, 10, 11). In addition, CAPS also contribute to SV exocytosis, as CAPS knockout neurons show markedly reduced docked SVs and stimulus-evoked exocytosis (12, 13). This evidence indicates that CAPS are required for the release of DCVs and SVs.

Mammals express two isoforms, CAPS-1 and CAPS-2 (14), each containing five stored function domains: dynactin1-interacting domain (DID), which mediates vesicle sorting (15), C_2_–PH module, constituting C_2_ and PH domains, which binds PI(4,5)P2 membrane (13, 16, 17), DAMH domain, which interacts with SNARE proteins (18, 19), a C-terminal DCV domain appears to be important for large-dense core vesicle binding (Fig. 1*A*) (17, 20). Vesicle exocytosis was severely impaired when mutations or truncations were induced in these domains (21, 22). Intriguingly, a recent study demonstrated that strong overexpression of a naturally occurring CAPS-2 splice variant, comprising solely DID, C_2_, and PH domains, partially rescued the transmitter release deficit in cultured CAPS double-KO neurons and adrenal chromaffin cells (23). However, the precise mechanisms by which the DID, C_2_, and PH domains jointly prime vesicles for release without the C-terminal half of CAPS, however, remain enigmatic and need to be determined. The crystal structure of the CAPS-1 C_2_–PH module has been solved (13), and a tight binding mode between C_2_ and PH tandem has been revealed. Upon tight interaction, C_2_ and PH constitute an effective unit with enhanced PI(4,5)P2 binding ability, which is essential for CAPS function in Ca^2+^-dependent exocytosis. In spite of these properties, no structural information about the DID has been reported, which hinders our understanding of the molecular mechanism of CAPS in vesicle exocytosis.

**Figure 1:**
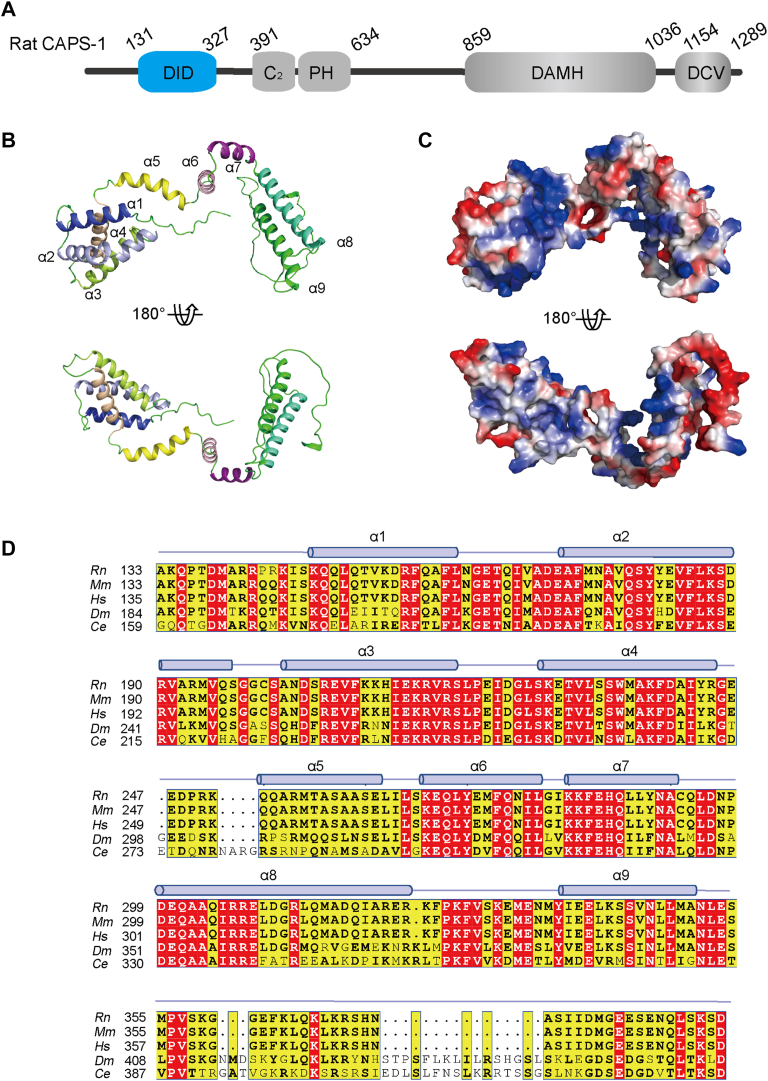
**Structure prediction of the rat CAPS-1 DID.***A*, schematic diagrams of CAPS-1. CAPS-1 contains the DID, C_2_, PH, DAMH, and DCV domains. The DID is shown in *blue*. *B*, the structure of DID predicted by AlphaFold2 (residues 133–391) is shown in two orientations rotated ∼180° with respect to each other. The α-helix are labeled with the corresponding numbers. *C*, electrostatic potential at the surface of the DID. The electrostatic potential was calculated by generating the local protein contact potential (PyMOL) and scaled from −5 kT/e (electron) to 5 kT/e, with *red* and *blue* denoting negative and positive potential, respectively. *D*, sequence alignment of DID residues in CAPS orthologs from *Rn* (*Rattus norvegicus*), *Mm* (*Mus musculus*), *Hs* (*Homo sapiens*), *Dm* (*Drosophila melanogaster*), and *Ce* (*Caenorhabditis elegans*). Numbering and secondary structural elements (*column* represents α-helix, *dotted line* represents linker) are shown. CAPS, Ca^2+^-dependent activator protein for secretion; DCV, dense core vesicle; DID, dynactin1-interacting domain.

In this study, we analyzed the structure of rat CAPS-1 DID using AlphaFold2. The predicted structure illustrated many charged residues loaded on the outer surface of the DID. We revealed a plasma membrane binding for the role of DID and found that DID clusters phosphatidylserine (PS)-containing liposomes. In addition, the DID plays a key role in stimulating liposome fusion; lacking the DID leads to remarkably reduced vesicle secretion in PC12 cells. Collectively, our data suggest that the DID of CAPS is an effective plasma membrane–binding domain to promote vesicle secretion.

## Results

### Structural prediction of the rat CAPS-1 DID

The CAPS-1 DID was considered the central region responsible for vesicle sorting (15). Moreover, a recent study revealed that overexpression of a splice variant, comprising solely the DID, C_2_–PH module, was sufficient to partially rescue the transmitter release deficit in cultured CAPS double-KO neurons and adrenal chromaffin cells (23). These findings suggest that the N-terminal portion of CAPS-1, including the DID, harbors intrinsic functionality important for exocytosis. While structural and functional roles of the C_2_–PH module have been extensively characterized, the DID remains poorly understood, and no high-resolution structural data have been reported to date.

To analyze the structure of the CAPS-1 DID, we downloaded the structure of rat CAPS-1 Q62717-F1-v4) from the AlphaFold database, from which the structure of the CAPS-1 DID (residues 133–391) was extracted and then subjected to structural analysis (Fig. 1, *B* and *C*). Sequence alignment revealed a strong evolutionary conservation across species (Fig. 1*D*), highlighting the likely functional significance of DID. The predicted model demonstrates an elongated conformation composed of nine *α-helices* (H1–H9) connected by short loops and flexible turns, forming a dynamic structural framework (Fig. 1*B*). The architecture features two distinct lobes: a tightly packed N-terminal subdomain formed by helices H1–H4 and a parallel-oriented C-terminal lobe comprising helices H8 and H9. These subdomains are interconnected through helices H5–H7, which creates a semiflexible hinge region. This central hinge exhibits potential conformational plasticity, suggesting a mechanism for domain reorientation that may facilitate molecular interactions essential to its biological role. We noted that the predicted aligned error plot indicated a coprediction between the DID and the region (residues 648–925) of CAPS-1 (Fig. S1). To determine whether the purified standalone CAPS-1 DID protein is correctly folded, we subjected it to CD spectroscopy and size-exclusion chromatography (SEC) analyses. The results demonstrated that the purified DID protein adopts an α-helical structure and exists as a stable, homogeneous species in solution (Fig. S2).

The predicted model revealed a striking predominance of hydrophilic residues bearing positive charges on the surface of DID (Fig. 1*C*). This distinctive electrostatic profile implies significant biological relevance, as it positions the domain to engage with anionic biomolecules through charge complementarity, including interactions with negatively charged phospholipid bilayers or acidic regions within protein domains. This electrostatic profile hints at a functional role in mediating membrane association or protein–protein interactions during vesicle docking and priming stages of exocytosis.

### DID binds to PS-containing liposomes

The DID of CAPS-1 features a surface enriched with hydrophilic and positively charged residues, a characteristic that has not been previously reported. Given the established role of CAPS as a vesicle tethering factor that interacts with the plasma membrane, we investigated whether the DID could mediate membrane binding.

**Figure 2:**
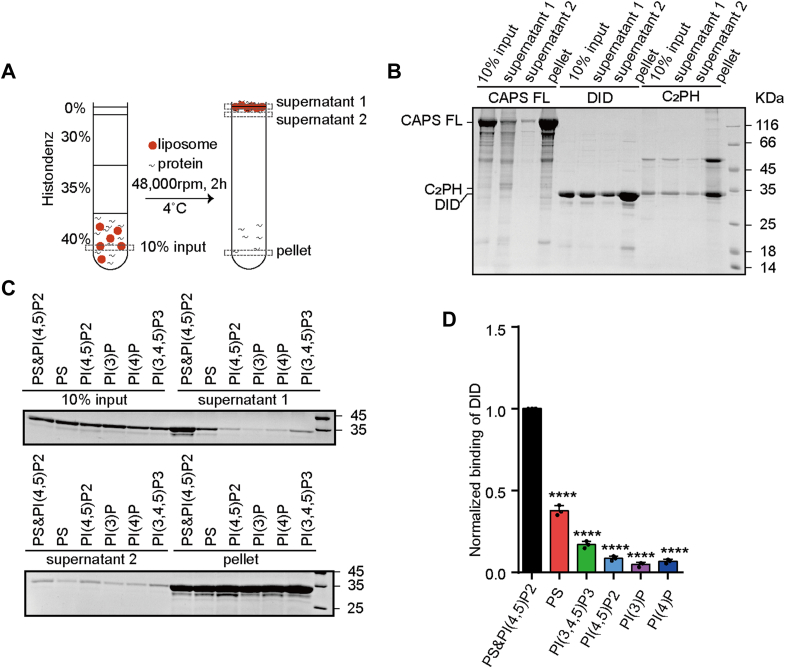
**The DID binds to phosphatidylserine-containing liposomes.***A*, schematic diagram of the liposome coflotation assay. After centrifugation, liposomes (*red*) and bound proteins (*black*) floated to the *top* of the density gradients, whereas unbound proteins remained at the *bottom*. *B*, comparison of the binding of the full-length CAPS-1, DID, and C_2_PH module to the liposomes. Proteins were coflotated with liposomes composed of POPC:POPE:DOPS:PIP2 (58:20:20:2). A representative gel from one of three independent experiments is shown. *C*, specificity of the CAPS DID for phospholipid binding. The DID was cofloated with liposomes composed of POPC:POPE:DOPS (60:20:20) or POPC:POPE:DOPS (58:20:20) supplemented with the indicated 2% phosphoinositides. *D*, quantification of the binding of the DID. Data are processed by ImageJ (National Institutes of Health) and presented as the mean ± SEM (n = 3, technical replicates). The intensity of the bound protein in the presence of PI(4,5)P2 was normalized to 1. CAPS, Ca^2+^-dependent activator protein for secretion; DID, dynactin1-interacting domain; DOPS, 1,2-dioleoyl-*sn*-glycero-3-phosphoserine; PI(4,5)P2, phosphatidylinositol 4,5-bisphosphate; POPC, 1-palmitoyl-2-oleoyl-*sn*-glycero-3-phosphocholine; POPE, 1-palmitoyl-2-oleoyl-*sn*-glycero-3-phosphoethanolamine.

To test this, we performed liposome coflotation assays using synthetic liposomes that mimic the lipid composition of the plasma membrane (58% phosphatidylcholine:20% phophatidylethanolamine (PE):20% PS:2% PI(4,5)P2) (Fig. 2*A*). The full-length CAPS-1, isolated C_2_–PH module, and DID all bind to these liposomes (Fig. 2*B*). Therefore, we hypothesized that the CAPS DID was another membrane-binding domain that may be important for CAPS function. To further characterize the binding specificity of DID, we prepared liposomes containing 2% PI(3)P, PI(4)P, PI(4,5)P2, or PI(3,4,5)P3, respectively. The coflotation assays revealed that the DID exhibited no clear preference for specific phosphoinositide headgroups (Fig. 2, *C* and *D*). Intriguingly, quantitative analysis revealed that the membrane binding strength was positively correlated with the surface charge density (Fig. 2*D*), demonstrating a charge-driven rather than a ligand-specific binding mechanism.

These findings support the hypothesis that the DID functions as a general membrane-anchoring module, likely through charge-based interactions with anionic phospholipids, such as PS or PIP species. This property may facilitate CAPS localization to the plasma membrane and promote its role in exocytosis.

### DID clusters liposomes

**Figure 3:**
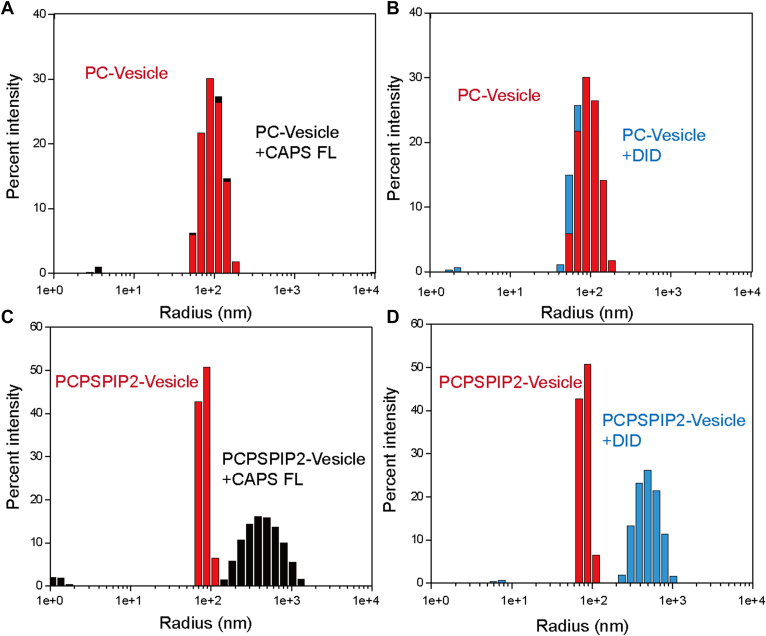
**CAPS-1 DID clusters PS-containing liposomes.***A*–*D*, the particle size in samples containing phospholipid vesicles alone *(red bars*) or after incubation with protein for 5 min (*blue/black bars)* were measured using DLS. The liposomes had a standard lipid composition, either without PS–PIP2 (*A*, *B*) or supplemented with PS and PIP2 (*C*, *D*). All experiments were performed in three independent replicates. CAPS, Ca^2+^-dependent activator protein for secretion; DID, dynactin1-interacting domain; DLS, dynamic light scattering; PS, phosphatidylserine.

During liposome coflotation assays, we observed slight turbidity upon mixing the CAPS-1 DID with liposomes, raising the possibility that the DID might induce vesicle aggregation. To directly assess whether the CAPS-1 DID promotes liposome clustering, we employed dynamic light scattering (DLS) to monitor changes in particle size distribution. DLS quantification demonstrated that the DID and full-length CAPS-1 failed to cluster plain liposomes lacking PS (Fig. 3, *A* and *B*). Notably, neither CAPS-1 nor the DID protein alone formed aggregates in the absence of liposomes, indicating that self-aggregation does not account for the observed clustering activity (Fig. S3, *A* and *B*). Intriguingly, dramatic increases in particle size observed by DLS revealed efficient clustering of PS/PI(4,5)P2-containing vesicles caused by the DID and full-length CAPS-1 (Fig. 3, *C* and *D*). Furthermore, control experiments confirmed that glutathione-*S*-transferase (GST) protein did not induce clustering of either plain liposomes or PS-containing vesicles (Figs. S3, *C* and *D*), underscoring the specificity of the DID for acidic phospholipids. To further corroborate the vesicle clustering induced by the CAPS-1 DID, we performed transmission electron microscopy to directly visualize liposome aggregation. Consistent with DLS results, GST control protein did not induce clustering of any liposomes tested. In contrast, DID and full-length CAPS-1 promoted prominent aggregation of vesicles (Fig. S4).

### CAPS-1 DID stimulates lipid mixing

**Figure 4:**
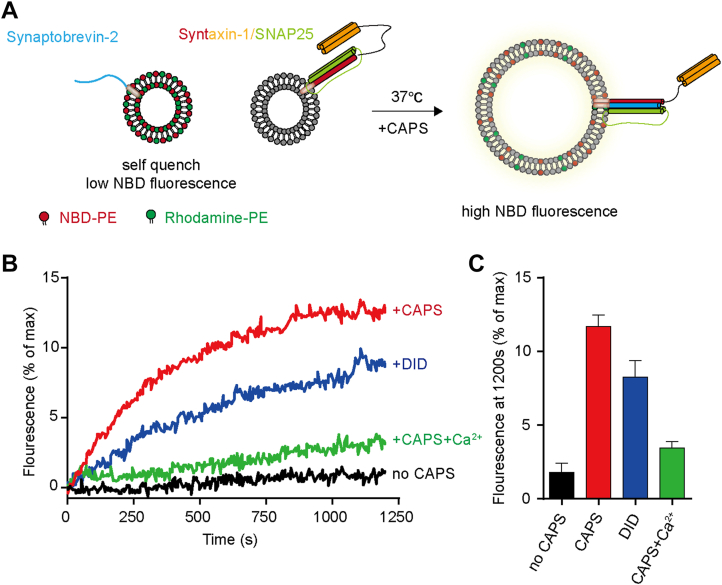
**CAPS-1 DID stimulates lipid mixing.***A*, schematic diagram of the lipid mixing of liposomes containing the Syx1–SN25 complex and PIP2 with liposomes containing Syb2 (1–116). NBD-PE and Rhodamine-PE were incorporated into Syb2 liposomes. NBD fluorescence emission was monitored at 538 nm. *B*, effects of the DID and full-length CAPS-1 on lipid mixing, with quantification shown in (*C*). Full-length CAPS-1 (1.5 μM) and the DID (20 μM) were applied. Data are presented as mean ± SEM; n = 3 technical replicates. Values that differ significantly are indicated (∗*p* < 0.05, ∗∗∗*p* < 0.001, and ∗∗∗∗*p* < 0.0001 two-tailed *t* test). CAPS, Ca^2+^-dependent activator protein for secretion; DID, dynactin1-interacting domain; NBD, *N*-7-nitrobenz-2-oxa-1,3-diazol-4-yl; PE, phophatidylethanolamine.

The membrane remodeling capacity of Munc13/Syt1 C_2_AB domains to cluster liposomes underpins their catalytic potency in accelerating SNARE-mediated membrane fusion (24, 25). Hence, we hypothesize that the CAPS DID is also able to stimulate SNARE-dependent lipid mixing. To rigorously validate this mechanistic postulate, we employed a reconstituted FRET assay system quantifying CAPS-accelerated SNARE-catalyzed membrane fusion efficiency (Fig. 4*A*). Consistent with previous results, CAPS-1 promoted lipid mixing between liposomes containing the Syx1–SN25 complex and liposomes containing Syb2 (Fig. 4, *B* and *C*). Notably, the CAPS-1 DID also significantly enhance SNARE-dependent lipid mixing, although the magnitude of this effect was relatively modest compared with full-length CAPS-1. In contrast, GST protein did not promote vesicle fusion, whereas the Syt C_2_B domain significantly enhanced lipid mixing under the same conditions (Fig.S5). This attenuated effect likely reflects the functional importance of other domains in CAPS-1 during vesicle secretion. For instance, the DAMH domain of CAPS-1 has been shown to directly facilitate SNARE complex assembly. Thus, the DID may act synergistically with other CAPS-1 regions to promote efficient exocytosis.

### Functional analysis of CAPS-1 DID in DCV exocytosis in PC12 cells

**Figure 5:**
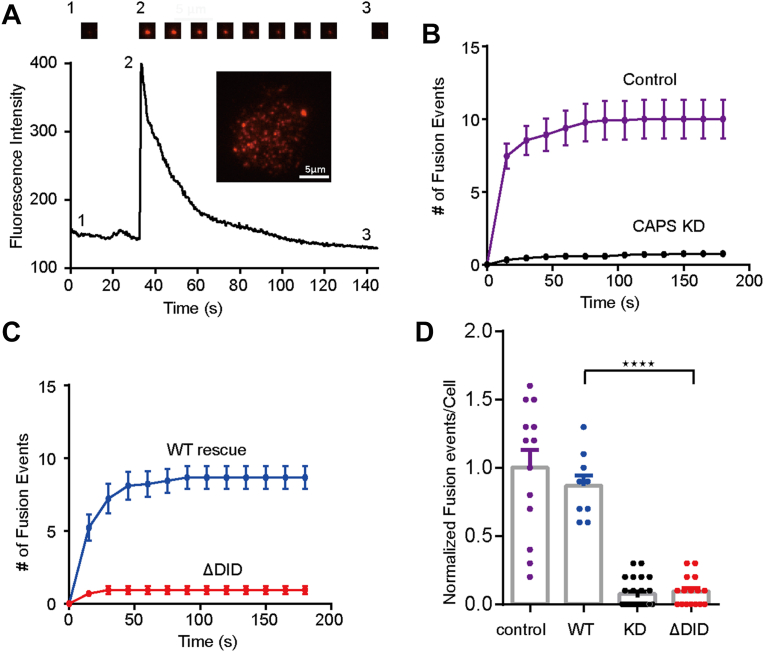
**Functional analysis of CAPS-1 DID in exocytosis.***A*, representative TIRF image of PC12 cells expressing NPY-td-mOrange2. Fluorescence intensity changes for a single DCV fusion event are shown. *B* and *C*, rescue studies show varying degrees of impairment in DCV exocytosis caused by the mutations. DCV exocytosis was evoked by depolarization with high-K^+^ stimulation at time 0. The *curves* indicate the cumulative number of fusion events per cell. The cumulative number of fusion events in WT PC12 cells (control cells) was normalized to 1. *D*, the number of cumulative fusion events observed at 180 s in (*B*, *C*). Fusion events in WT PC12 cells (control cells) were set to 1. Values that differ significantly are indicated (∗∗∗∗*p* < 0.0001; ns, not significant; two-tailed *t* test). Data are presented as mean ± SEM; n = 9 to 50 biological replicates. Values that differ significantly are indicated (two-tailed *t* test). CAPS, Ca^2+^-dependent activator protein for secretion; DCV, dense core vesicle; DID, dynactin1-interacting domain; TIRF, total internal reflection fluorescence.

To investigate the regulatory role of the DID in CAPS-1-mediated Ca^2+^-dependent DCV exocytosis, we conducted systematic functional assays in PC12 cells. Vesicle release was induced through conventional potassium ion stimulation (26), which depolarizes PC12 cells to promote calcium influx. Single-vesicle exocytotic dynamics were monitored *via* total internal reflection fluorescence microscopy by tracking NPY-td-mOrange2 fluorescence intensity surges at the plasma membrane contact zone (Fig. 5*A*), following established protocols (12, 27).

RNA interference–mediated suppression of CAPS-1 expression (*via* shRNA knockdown) resulted in a marked decrease in calcium-triggered secretory events, replicating prior observations (28). Reconstitution experiments demonstrated that wildtype CAPS-1 expression restored exocytotic activity, whereas the DID-truncated mutant exhibited compromised functional rescue (Fig. 5, *B*–*D*). Notably, the subcellular localization and expression level of the truncated protein remained unchanged (Fig.S6). Furthermore, protein expression and SEC analyses confirmed that deletion of the DID did not alter the homogeneous aggregation state of CAPS-1 (Fig. S7). These *in vivo* observations align with our *in vitro* biochemical data, conclusively identifying the DID as a nonredundant functional element for CAPS-1-mediated Ca^2+^-triggered exocytosis.

## Discussion

Recently, significant advances have been made in characterizing the functions of the different domains of CAPS-1, particularly structural and functional characterization of its C_2_–PH and DAMH domains (12, 13, 29). These findings collectively highlight essential regulatory functions of CAPS-1 in vesicular trafficking and regulated exocytosis (30, 31). However, it was unclear how the DID of CAPS-1 is involved in vesicle fusion. Notably, previous studies identified that exogenous expression of the N-terminal region alone (containing the C_2_–PH unit and DID) could rescue vesicle secretion defects in CAPS-1 knockout mice during the early development stage (23). While the C_2_–PH module has been illustrated to mediate specific binding to PIP_2_-enriched membranes (13), the structural basis and mechanistic roles of the DID remain enigmatic. The DID was initially suggested to be a dynactin-interacting domain (15). Indeed, experimental evidence has revealed that binding of the DID to dynactin can be ascertained through assays like yeast two-hybrid and immunoprecipitation; the DID has also been implicated in depression-related pathologies (15). However, its broader role in vesicle dynamics and exocytosis had not been clearly defined.

**Figure 6:**
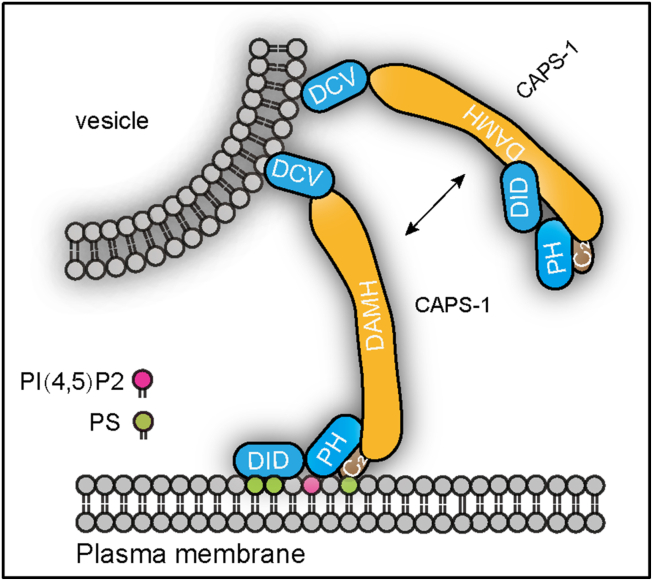
**A working model for CAPS-1 in vesicle tethering.** CAPS-1 orchestrates vesicle tethering through coordinated interactions among its modular domains: Under regulatory conditions, the DID disengages from its cofolding interactions with the C-terminal region (1). The DID achieves high-affinity anchoring to the plasma membrane *via* electrostatic interactions (2); The C2–PH functional unit selectively targets PIP2-enriched membrane microdomains, thereby ensuring spatial precision; and (3) the DCV domain directly engages secretory vesicles, forming a tripartite bridge that dramatically potentiates vesicle docking and fusion efficiency. CAPS, Ca^2+^-dependent activator protein for secretion; DCV, dense core vesicle; DID, dynactin1-interacting domain.

Based on the structural prediction from the AlphaFold database, we systematically analyzed the structure of the CAPS-1 DID (Fig. 1). The predicted architecture comprises stacked α-helices forming an exposed surface enriched with positive charges, suggesting potential for charge-mediated molecular interactions (Fig. 1*C*). Given the electronegative nature of plasma membranes, we performed protein–liposome binding assays to demonstrate that DID–membrane association occurs through nonselective electrostatic interactions rather than specific phospholipid recognition (Fig. 2). To investigate the functional implications of this nonspecific binding, we conducted vesicle aggregation assays under physiomimetic lipid conditions. Remarkably, DID alone recapitulated the vesicle-clustering activity typically requiring full-length CAPS-1 (Fig. 3). This membrane-tethering capacity was further corroborated by enhanced vesicle fusion in reconstituted liposome assays (Fig. 4). Physiological validation through CAPS-1 knockdown in PC12 cells revealed that DID-deficient mutants failed to rescue secretion defects, confirming the essential role of DID in vesicular release (Fig. 5). Based on these findings, we propose that CAPS-1 enhances vesicle secretion by mediating vesicle tethering (Fig. 6). Specifically, CAPS-1 orchestrates this process through the coordinated interactions across its modular domains: under regulated conditions, DID disengages from its cofolding with the C-terminal region (1). The DID achieves a high-affinity anchoring to the plasma membrane *via* electrostatic interactions (2). The C_2_–PH functional unit selectively targets PIP2-enriched membrane microdomains, ensuring spatial precision (3). The DCV domain directly engages secretory vesicles, forming a tripartite bridge that dramatically potentiates vesicle docking and fusion efficiency. This domain-specific synergy enables CAPS-1 to function as a molecular scaffold, synchronizing membrane recognition, vesicle capture, and secretion machinery assembly to drive robust neurotransmitter or hormone release.

Although our findings establish that the DID facilitates vesicle secretion through a charge-dependent membrane anchoring, critical questions remain regarding the functional interplay between the DID and the adjacent domain of CAPS. In the full-length structural prediction of CAPS-1 (Fig. S1), the DID appears to be cofolded with the DAMH domain. Although such predictions are based on *in silico* models of isolated protein and may not fully reflect the physiological structure of CAPS-1 during function, investigating the conditions under which this cofolding might be disrupted remains a meaningful direction for understanding DID functionality. Second, our study demonstrates that the CAPS-1 DID binds the negatively charged phospholipids, whereas previous work has shown that the C_2_–PH module of CAPS-1 selectively interacts with PIP2 on the plasma membrane. Whether there is synergy and temporal coordination between membrane binding mediated by the DID and that mediated by the C_2_–PH module constitutes another key avenue for future research. Furthermore, it will be important to examine whether the cofolding between the DID and DAMH domains modulates DAMH function, thereby potentially enabling intramolecular regulation of CAPS-1 activity. Further studies will be essential to dissect the dynamic interplay between these domains and their coordinated roles in membrane targeting and exocytotic efficiency.

## Experimental procedures

### DNA constructs and protein purification

CAPS DID was constructed into pGEX-6p-1 vector (GE Healthcare) incorporating an N-terminal PreScission protease-cleavable GST tag. The recombinant GST fusion protein was expressed in *Escherichia coli* BL21 (DE3) strain cultured in LB medium at 37 °C to an absorbance of 0.6 to 0.8 at 600 nm and was induced with 0.4 mM IPTG at 20 °C for 20 h. After centrifugation at 2930*g* for 30 min, cells were resuspended in a buffer containing 50 mM Tris (pH 8.4) and 1 M NaCl. Cells were broken using an ultrasonic homogenizer (Scientz-IID). Cell lysates were centrifuged at 30,966*g* for 30 min at 4 °C. The supernatants were incubated with 2 ml glutathione-Sepharose beads (Amersham-Pharmacia Biotech) at 4 °C for 3 h. The bound proteins were washed with PBS (137 mM NaCl, 2.7 mM KCl, 10 mM Na_2_HPO_4_, 2 mM KH_2_PO_4_, pH 7.4) with 750 mM NaCl. The GST tag was cleaved from the proteins by GST-fused PreScission protease at 4 °C overnight on the beads. The proteins were further purified by SEC on Superdex 75 pg 16/600 column (GE Healthcare) and stored in 25 mM Hepes (pH 7.4), 150 mM KCl, and 10% glycerol (v/v).

The plasmid encoding NPY-td-mOrange2 was purchased from Addgene (plasmid no.: 83497). Complementary DNA encoding full-length rat CAPS-1 (residues 1–1289) was cloned into the pEGFP-N3 vector (Clontech) using EcoRI and BamHI. The CAPS-1 shRNA (28) was cloned into pFHUUIG_shortU6 (l309) plasmid downstream of the H1 promoter by using the restriction enzyme sites XhoI and XbaI; the CAPS-1 wildtype and mutant rescue sequences were cloned into pFHUUIG_shortU6 (l309) plasmid after the ubiquitin promoter by using the restriction enzyme sites BamHI and EcoRI.

The plasmid pFastBac-CAPS-1 for eukaryotic expression was generated by subcloning CAPS-1 from a pcDNA3.1-CAPS-1 plasmid into pFastBacHtB vector (Invitrogen), which contains a polyhedron promoter (for high-level expression of recombinant protein in insect cells) before the start codon and encodes an N-terminal tobacco etch virus–cleavable 6xHis-tag. Full-length CAPS-1 was expressed in Sf9 insect cells as described previously (27). Sf9 insect cells were infected with the CAPS-1 baculovirus, harvested about 72 h postinfection, and resuspended in lysis buffer (50 mM Tris [pH 8.4], 300 mM NaCl, and 5 mM imidazole). Cells were lysed and then centrifuged at 30,966*g* for 30 min, and the supernatant was incubated with Ni^2+^–NTA agarose (QIAGEN) at 4 °C for 2 to 3 h. The beads were washed with lysis buffer supplied with additional 10 to 30 mM imidazole. The protein was eluted with 300 mM imidazole and further purified by gel filtration in a buffer containing 25 mM Hepes (pH 7.4), 150 mM KCl, and 10% glycerol (v/v).

### Liposome preparation

Lipid powder (all from Avanti Polar Lipids) was dissolved in chloroform at a concentration of 10 mg/ml for storage at −20 °C, except for brain PI(4,5)P2 (derived from porcine brain) in chloroform:methanol:water (20:9:1) at 1 mg/ml. Lipids were mixed at the proper ratio as indicated in the figures or legends to a final concentration of 5 mM and dried under nitrogen followed by vacuum for at least 3 h. Lipid films were dissolved in buffer A (25 mM Hepes [pH 7.4], 150 mM KCl, and 10% glycerol [v/v]) with liquid nitrogen. Dissolved lipids were then extruded through a 200 nm polycarbonate film (Whatman) using a miniextruder (Avanti Polar Lipids). The prepared liposomes were checked using DLS on a DynaPro Nanostar (Wyatt Technology) before usage.

### Liposome coflotation assay

Liposome (2 mM total lipids) compositions are indicated in the figures or legends. Liposomes were incubated with 15 μM proteins in buffer A (unless stated otherwise) for 1 h at room temperature. Liposomes and bound proteins were isolated by flotation on a Histodenz (Sigma) density gradient (40%:35%:30%) using an S50ST rotor (Eppendorf CS150FNX) at 270,000*g* for 2 h. Samples on the top (40 μl, S1) and the second (S2) were taken and analyzed by SDS-PAGE and Coomassie Brilliant Blue staining.

### Lipid mixing experiments

All lipids were purchased from Avanti Polar Lipids. For lipid mixing experiments starting with the Syx1 (1–288)–SN25 complex, liposomes reconstituted with Syb2 contained 60% 1-palmitoyl-2-oleoyl-*sn*-glycero-3-phosphocholine, 17% 1-palmitoyl-2-oleoyl-*sn*-glycero-3-phosphoethanolamine, 20% 1,2-dioleoyl-*sn*-glycero-3-phosphoserine, 1.5% Rhodamine-PE, 1.5% *N*-7-nitrobenz-2-oxa-1,3-diazol-4-yl (NBD)–PE, and liposomes reconstituted with the Syx1–SN25 complex contained 60% 1-palmitoyl-2-oleoyl-*sn*-glycero-3-phosphocholine, 18% 1-palmitoyl-2-oleoyl-*sn*-glycero-3-phosphoethanolamine, 20% 1,2-dioleoyl-*sn*-glycero-3-phosphoserine, 2% PIP2, with a protein/lipid ratio of 1:500 and 1:800, respectively. The final concentrations of total lipids were 5 mM. Syb2 liposomes (0.25 mM lipids) were mixed with Syx1–SN25 liposomes (0.5 mM lipids) in the presence of 1.5 μM CAPS-1 or 20 μM DID in a total volume of 60 μl.

NBD fluorescence emission at 538 nm (excitation at 460 nm) was monitored with SpectraMAX i3x. All experiments were performed at 30 °C. At the end of each reaction, 1% CHAPS (w/v) was added to solubilize the liposomes. Each experiment was repeated at least three times. In lipid mixing, NBD fluorescence was calculated according to *E* = 100% × (*F*_obs_–*F*_0_)/(*F*_max_–*F*_0_), where *F*_obs_ is the observed fluorescence intensity and *F*_0_ is the initial intensity, and *F*_max_ is the observed fluorescence intensity upon detergent addition.

### Cell culture and transfection

PC12 cells were cultured in RPMI1640 medium (GIBCO) supplemented with 10% fetal bovine serum (GIBCO) at 37 °C in a 5% CO_2_ atmosphere at constant humidity. PC12 cells (grown to ⁓80% confluence in a 10 cm dish) were suspended in 0.3 ml of Electroporation Buffer (50 mM KH_2_PO_4_, 20 mM CH_3_COOK, 20 mM KOH, 5 mM MgSO_4_, pH 7.4) and transfected with 10 to 20 μg CAPS-1 shRNA plasmid or CAPS-1 shRNA and CAPS-1 WT/mutant rescue plasmid. Cells were plated on poly-l-lysine-coated 20-mm glass-bottom dishes (NEST) for 48 to 60 h prior to total internal reflection fluorescence analysis.

### Sequence alignment

Sequence alignment was performed with Clustal Omega (32) and analyzed with ESPript 3.0 (33).

### Quantification and statistical analysis

Data compilation and statistical analysis were performed using Prism 6.01 (GraphPad) with significance value as ∗*p* < 0.05; ∗∗*p* < 0.01; ∗∗∗*p* < 0.001; ∗∗∗∗*p* < 0.0001; ns, no significant difference. Two-tailed *t* test was performed in the statistical analyses, and all data were reported as mean ± SD. The *p* values and replicates with the definition of n for all experiments can be found in the figure legends. All experiments were performed at least three times independently (or on at least three separate cells) to ensure rigor and reproducibility.

## Data availability

The data that support the findings of this study are available from the corresponding author upon request.

## Supporting information

This article contains supporting information.

## Conflict of interest

The authors declare that they have no conflicts of interest with the contents of this article.

## References

[1] Südhof T.C. (2014). The molecular machinery of neurotransmitter release (Nobel lecture). Angew. Chem. Int. Ed. Engl..

[2] Südhof T.C., Rizo J. (2011). Synaptic vesicle exocytosis. Cold Spring Harb Perspect. Biol..

[3] Jahn R., Cafiso D.C., Tamm L.K. (2024). Mechanisms of SNARE proteins in membrane fusion. Nat. Rev. Mol. Cell Biol..

[4] James D.J., Martin T.F. (2013). CAPS and Munc13: CATCHRs that SNARE vesicles. Front Endocrinol. (Lausanne).

[5] Stevens D.R., Rettig J. (2009). The Ca(2+)-dependent activator protein for secretion CAPS: do I dock or do I prime?. Mol. Neurobiol..

[6] Zhang L., Wei Z., Dai Y., He F., Sun T. (2025). The role of CAPS in Ca(2+)-regulated exocytosis: promotion of vesicle tethering, priming, and fusion. Neuropharmacology.

[7] Sarafian T., Aunis D., Bader M.F. (1987). Loss of proteins from digitonin-permeabilized adrenal chromaffin cells essential for exocytosis. J. Biol. Chem..

[8] Walent J.H., Porter B.W., Martin T.F. (1992). A novel 145 kd brain cytosolic protein reconstitutes Ca(2+)-regulated secretion in permeable neuroendocrine cells. Cell.

[9] Avery L., Bargmann C.I., Horvitz H.R. (1993). The Caenorhabditis elegans unc-31 gene affects multiple nervous system-controlled functions. Genetics.

[10] Renden R., Berwin B., Davis W., Ann K., Chin C.T., Kreber R. (2001). Drosophila CAPS is an essential gene that regulates dense-core vesicle release and synaptic vesicle fusion. Neuron.

[11] Cornell R., Cao W., Liu J., Pocock R. (2022). Conditional degradation of UNC-31/CAPS enables spatiotemporal analysis of neuropeptide function. J. Neurosci..

[12] Zhou H., Wei Z., Wang S., Yao D., Zhang R., Ma C. (2019). Structural and functional analysis of the CAPS SNARE-Binding domain required for SNARE complex Formation and exocytosis. Cell Rep..

[13] Zhang L., Li L., Wei Z., Zhou H., Liu H., Wang S. (2023). The C(2) and PH domains of CAPS constitute an effective PI(4,5)P2-binding unit essential for Ca(2+)-regulated exocytosis. Structure.

[14] Jockusch W.J., Speidel D., Sigler A., Sorensen J.B., Varoqueaux F., Rhee J.S. (2007). CAPS-1 and CAPS-2 are essential synaptic vesicle priming proteins. Cell.

[15] Sadakata T., Washida M., Iwayama Y., Shoji S., Sato Y., Ohkura T. (2007). Autistic-like phenotypes in Cadps2-knockout mice and aberrant CADPS2 splicing in autistic patients. J. Clin. Invest..

[16] Petrie M., Esquibel J., Kabachinski G., Maciuba S., Takahashi H., Edwardson J.M. (2016). The vesicle priming factor CAPS functions as a homodimer via C2 domain interactions to promote regulated vesicle exocytosis. J. Biol. Chem..

[17] Grishanin R.N., Klenchin V.A., Loyet K.M., Kowalchyk J.A., Ann K., Martin T.F. (2002). Membrane association domains in Ca2+-dependent activator protein for secretion mediate plasma membrane and dense-core vesicle binding required for Ca2+-dependent exocytosis. J. Biol. Chem..

[18] James D.J., Kowalchyk J., Daily N., Petrie M., Martin T.F. (2009). CAPS drives trans-SNARE complex formation and membrane fusion through syntaxin interactions. Proc. Natl. Acad. Sci. U. S. A..

[19] Daily N.J., Boswell K.L., James D.J., Martin T.F. (2010). Novel interactions of CAPS (Ca2+-dependent activator protein for secretion) with the three neuronal SNARE proteins required for vesicle fusion. J. Biol. Chem..

[20] Kabachinski G., Kielar-Grevstad D.M., Zhang X., James D.J., Martin T.F. (2016). Resident CAPS on dense-core vesicles docks and primes vesicles for fusion. Mol. Biol. Cell.

[21] van Keimpema L., Kooistra R., Toonen R.F., Verhage M. (2017). CAPS-1 requires its C2, PH, MHD1 and DCV domains for dense core vesicle exocytosis in mammalian CNS neurons. Sci. Rep..

[22] Miyake K., Ohta T., Nakayama H., Doe N., Terao Y., Oiki E. (2016). CAPS1 RNA editing promotes dense core vesicle exocytosis. Cell Rep..

[23] Nguyen Truong C.Q., Nestvogel D., Ratai O., Schirra C., Stevens D.R., Brose N. (2014). Secretory vesicle priming by CAPS is independent of its SNARE-binding MUN domain. Cell Rep..

[24] Quade B., Camacho M., Zhao X., Orlando M., Trimbuch T., Xu J. (2019). Membrane bridging by Munc13-1 is crucial for neurotransmitter release. Elife.

[25] Xue M., Ma C., Craig T.K., Rosenmund C., Rizo J. (2008). The Janus-faced nature of the C(2)B domain is fundamental for synaptotagmin-1 function. Nat. Struct. Mol. Biol..

[26] Lang T., Wacker I., Steyer J., Kaether C., Wunderlich I., Soldati T. (1997). Ca2+-triggered peptide secretion in single cells imaged with green fluorescent protein and evanescent-wave microscopy. Neuron.

[27] Grishanin R.N., Kowalchyk J.A., Klenchin V.A., Ann K., Earles C.A., Chapman E.R. (2004). CAPS acts at a prefusion step in dense-core vesicle exocytosis as a PIP2 binding protein. Neuron.

[28] Kabachinski G., Yamaga M., Kielar-Grevstad D.M., Bruinsma S., Martin T.F. (2014). CAPS and Munc13 utilize distinct PIP2-linked mechanisms to promote vesicle exocytosis. Mol. Biol. Cell.

[29] Nestvogel D.B., Merino R.M., Leon-Pinzon C., Schottdorf M., Lee C., Imig C. (2020). The synaptic vesicle priming protein CAPS-1 shapes the adaptation of sensory evoked responses in Mouse visual cortex. Cell Rep..

[30] Kreutzberger A.J.B., Kiessling V., Stroupe C., Liang B., Preobraschenski J., Ganzella M. (2019). In vitro fusion of single synaptic and dense core vesicles reproduces key physiological properties. Nat. Commun..

[31] Crummy E., Mani M., Thellman J.C., Martin T.F.J. (2019). The priming factor CAPS1 regulates dense-core vesicle acidification by interacting with rabconnectin3beta/WDR7 in neuroendocrine cells. J. Biol. Chem..

[32] Sievers F., Higgins D.G. (2021). The clustal Omega multiple alignment package. Methods Mol. Biol..

[33] Guillon C., Gouet P. (2025). FoldScript: a web server for the efficient analysis of AI-generated 3D protein models. Nucleic Acids Res.

