# Supplementary material for: The DID of CAPS-1 anchors plasma membrane to promote vesicle exocytosis
Source: J Biol Chem. 2025 Nov 4;301(12):110902. doi: 10.1016/j.jbc.2025.110902 (PMC12702071; doi:10.1016/j.jbc.2025.110902)
Supplement: Supplemental Figures [file mmc1.docx]

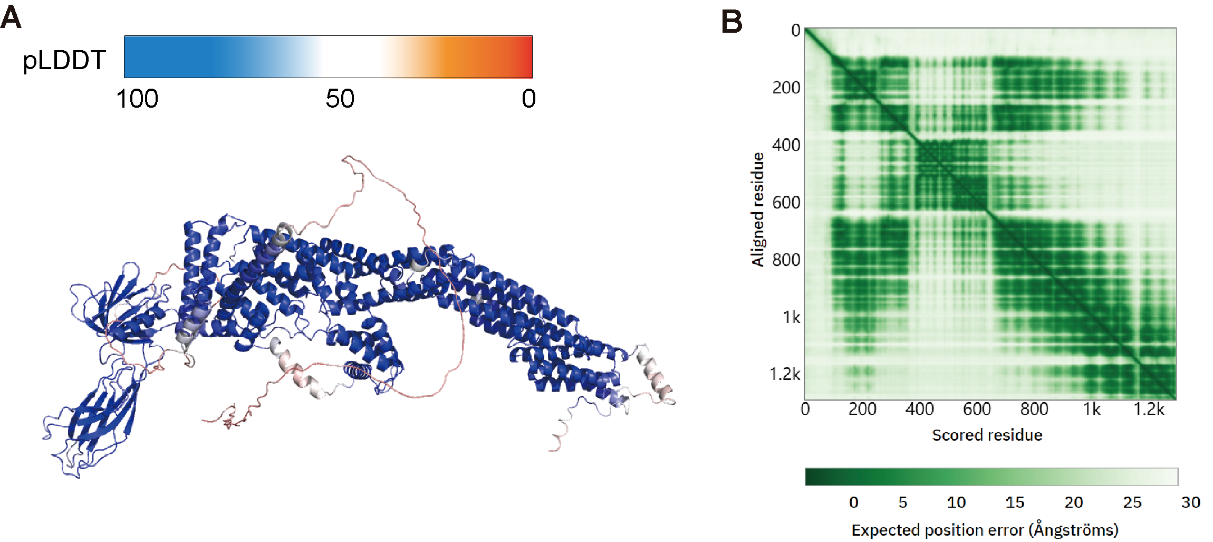


**Supplemental Figure 1:** The full-length structural model of CAPS-1. (**A**) Predicted three-dimensional structure of CAPS-1. The model is colored by pLDDT (per-residue confidence score), ranging from blue (high confidence, pLDDT = 100) through white (intermediate, pLDDT = 50) to red (very low confidence, pLDDT = 0). (**B**) Predicted Aligned Error (PAE) plot. The PAE matrix indicates the expected positional error (in Ångströms) between residues if the predicted model were aligned based on one residue pair.


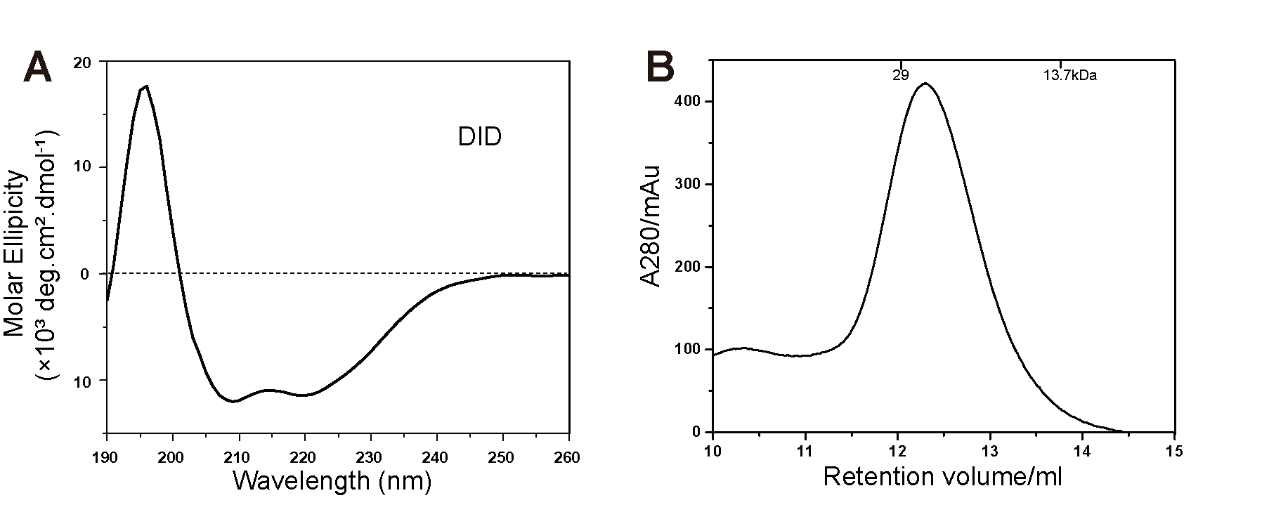


**Supplemental Figure 2:** The Circular Dichroism **(A)** and Size-Exclusion Chromatography **(B)** results for the DID domain demonstrate its stable, folded, and monodisperse nature in vitro.


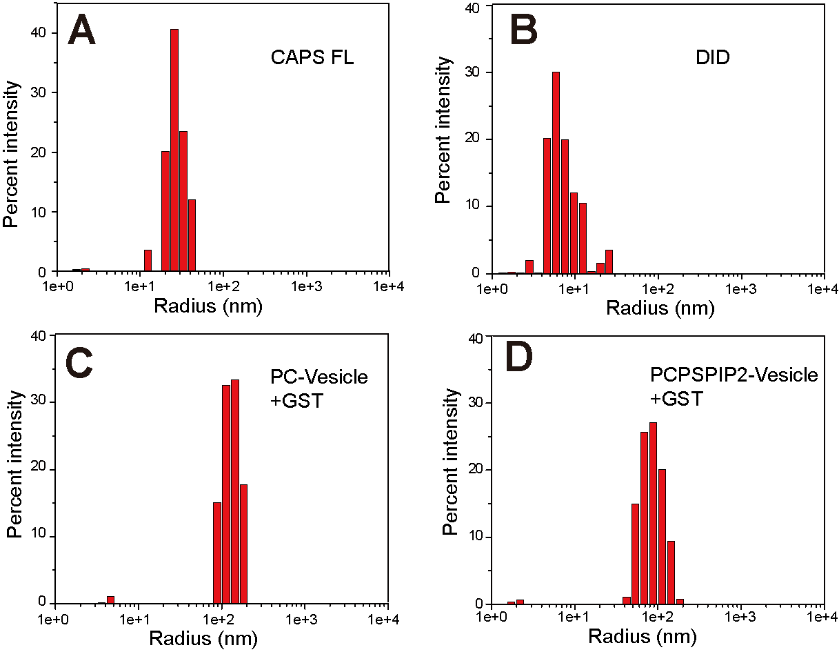


**Supplemental Figure 3:** The isolated CAPS-1 (**A**) and DID (B) proteins do not form large aggregates, and the GST control protein does not induce vesicle clustering(C, D). All experiments were independently repeated three times.


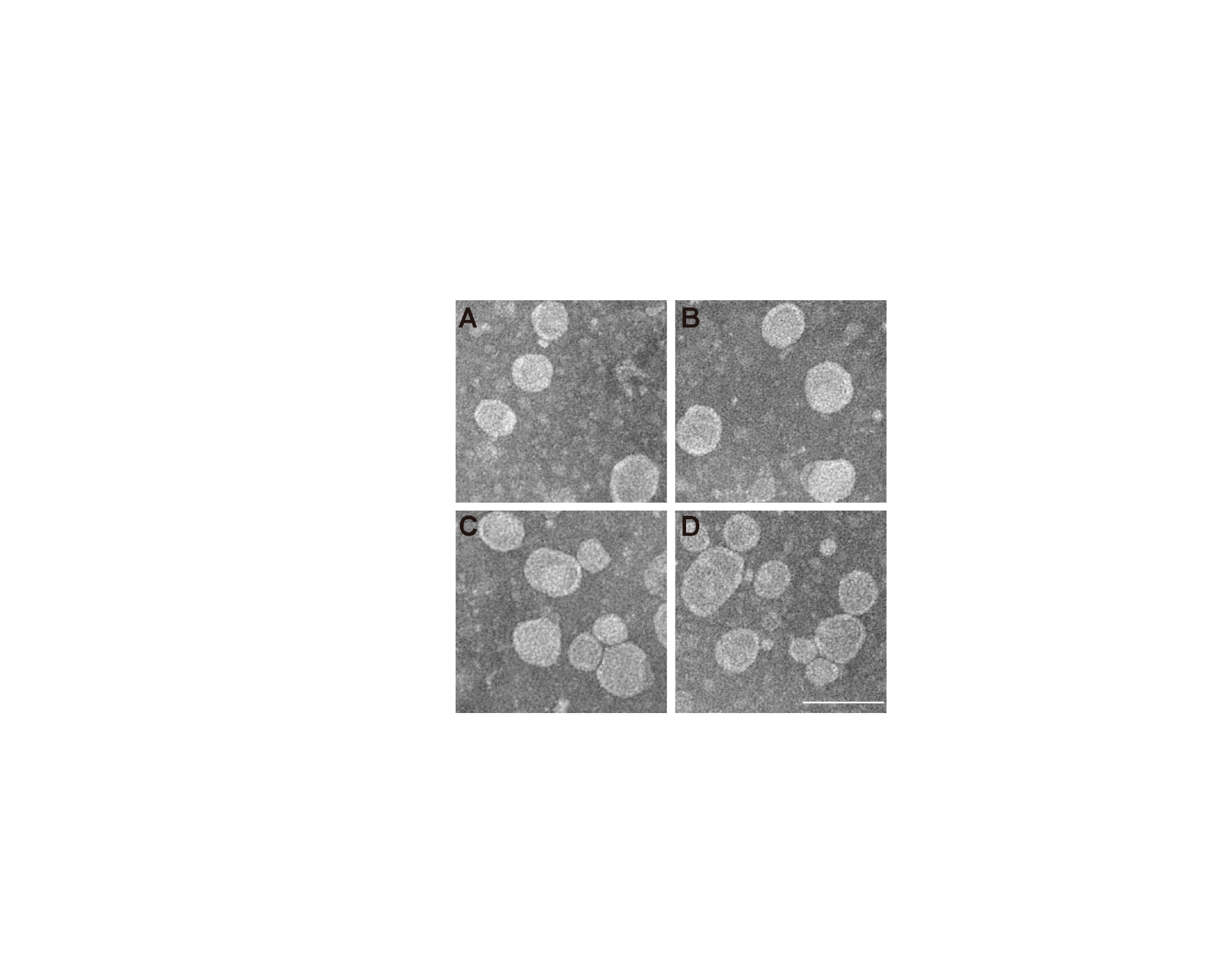


**Supplemental Figure 4:** Transmission electron microscopy (TEM) analysis of protein-induced vesicle clustering: (**A**) Vesicles alone (standard composition including PS); (**B**) Vesicles incubated with GST protein; (**C**) Vesicles incubated with DID protein; (**D**) Vesicles incubated with full-length CAPS-1 protein. Scale bar, 100 nm.


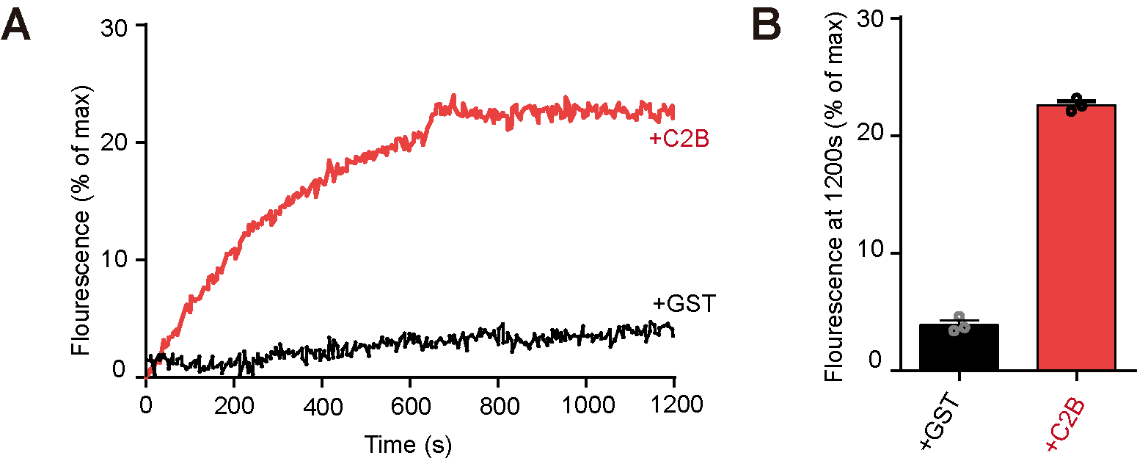


**Supplemental Figure 5:** Positive and negative controls for lipid mixing.

(A) Effects of the GST domain and Syt C_2_B on lipid mixing and quanti-fication of the results (B). Syt C_2_B (1.5 μM) and the GST (20 μM) were applied. Data are presented as mean ± SEM; n = 3 technical replicates.


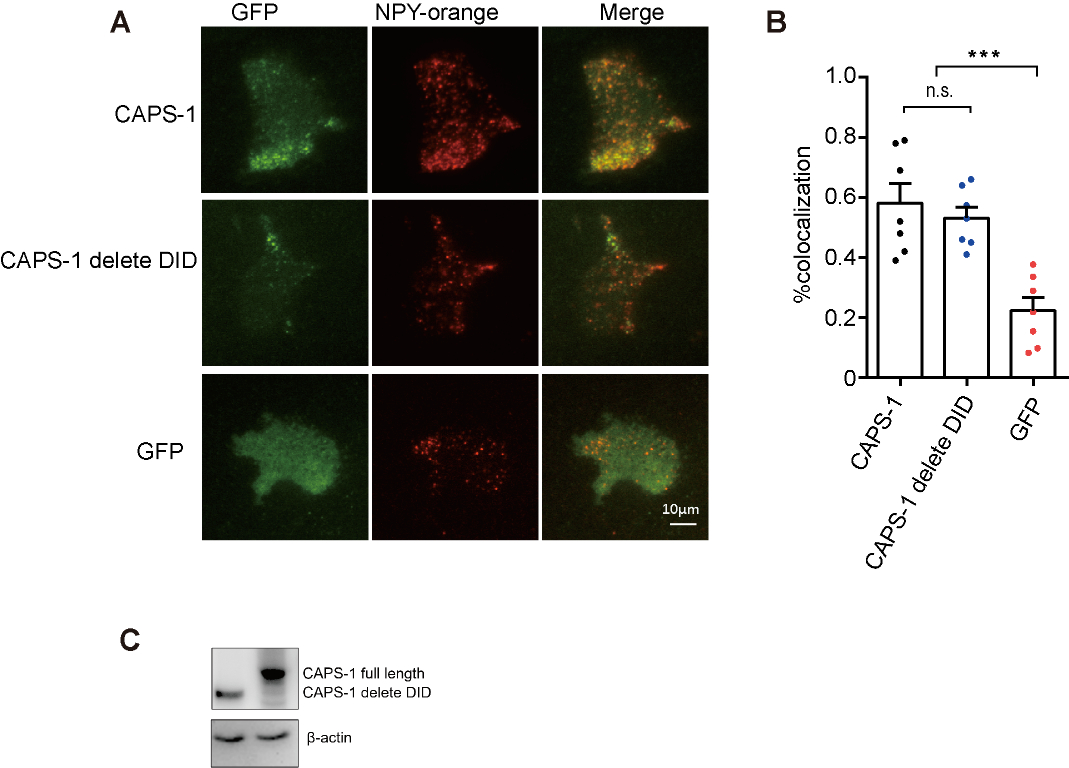


**Supplemental Figure 6:** Expression and subcellular localization of CAPS-1 and delete DID.(**A**) Representative TIRF image of PC12 cells expressing NPY-td-Orange2 and CAPS-1(or mutant). (**B**) Percentage of co-localization for DCVs and CAPS-1 (or the mutants). Data are presented as mean ± SEM; n = 7 biological replicates.Values that differ significantly are indicated (n.s.,no significant difference; two-tailed t test). (**C**) Western blot of the proteins expression in the functional assay.


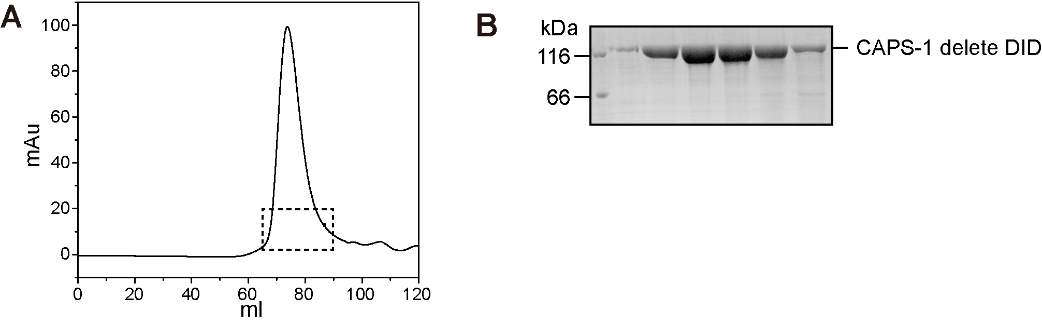


**Supplemental Figure 7:** Protein purification of CAPS-1 delete DID. **(A)** Gel filtration profiles on Superdex-200 of CAPS-1 delete DID. (**B**) Fractions corresponding to the peak maximum from size-exclusion chromatography were collected and analyzed by SDS-PAGE followed by Coomassie blue staining.
